# Intake of Food Supplements, Caffeine, Green Tea and Protein Products among Young Danish Men Training in Commercial Gyms for Increasing Muscle Mass

**DOI:** 10.3390/foods11244003

**Published:** 2022-12-11

**Authors:** Kirsten Pilegaard, Anne Sophie Majgaard Uldall, Gitte Ravn-Haren

**Affiliations:** National Food Institute, Technical University of Denmark, DK-2800 Kongens Lyngby, Denmark

**Keywords:** food supplements, coffee, (-)-epigallocatechin-3-gallate (EGCG), supplementation, adverse effects, safety, 1,3-dimethylamylamine, pre-workout (PWO) products, Jack3D, Craze

## Abstract

Sixty-three men (15–35 years of age) regularly training in Danish gyms and supplement users were interviewed about the use of supplemental protein and food supplements, intake of caffeine- and (-)-epigallocathechin-3-gallate (EGCG)-containing supplements and beverages and any experienced adverse effects. Protein powder (60%), fish oil (54%) and multivitamin/mineral supplements (41%) were the most popular products. The daily supplementary protein intake (mean 0.42 g/kg body weight, users only) in adult men contributed substantially to their protein intake and exceeded the recommended allowance (0.83 g/kg body weight) for six adult participants (14%). Thirty-eight percent of the adult men exceeded the daily caffeine intake presumed to be safe (400 mg) with coffee as the main contributor. Thirty percent drank green tea and among this percentage, two participants had an extreme daily intake (1.5 and 2 -L). EGCG intake could not be estimated from the food supplements due to the lack of label information. Eighteen participants (29%) reported having experienced adverse effects but seventeen did not consult a physician or report the adverse effect to the Danish food authority. The most common adverse effects were insomnia, shaking, headache and palpitations, itching of the skin and stinging. Pre-workout products accounted for 53% of the adverse effects. Three adverse effects came after intake of two brands of supplements known to have contained substances such as 1,3-dimethylamine or derivatives of phenylethylamines previously having caused serious adverse effects.

## 1. Introduction

In recent years, being a member of a gym has become popular in Denmark. An estimated 810,000 to 950,000 Danes were members of commercial gyms in 2015 compared with 550,000 in 2009, corresponding to around 15% of the total Danish population (5.7 million in 2016) [1,2]. The popularity is reflected in increasing accessibility to commercial gyms with a doubling in numbers from 334 in February 2007 to 663 in February 2016 [1,3]. Memberships of some gyms may include an all-in concept that guarantees the member one daily product to be consumed at the gym. The assortment includes different drinks, shakes, bars and shots fortified with vitamins, minerals, caffeine or protein. Besides the products available at the gym, numerous food or dietary supplements and protein products are sold in retail and specifically target athletes focusing on exercise performance, increasing muscle mass and improving endurance.

The European Union (EU) regulation defines food supplements ‘as foodstuffs having the purpose of supplementing the normal diet and which are concentrated sources of nutrients or other substances with a nutritional or physiological effect, alone or in combination, marketed in dose form and designed to be taken in measured small unit quantities’ [4]. The chemical sources of vitamins and minerals added to food supplements are regulated at the EU level; however, no common maximum levels of these compounds in food supplements have yet been set. For other nutrients and other substances with nutritional or physiological effects used as ingredients in food supplements, e.g., amino acids, essential fatty acids, fibers, various plants and herbal extracts, national rules may be applicable if no specific EU rules have been adopted [4]. The Danish regulation on food supplements requires a notification to the Danish Veterinary and Food Administration (DVFA) of all food supplements marketed in Denmark. The information that vendors are required to send to the DVFA includes the name of the food supplement, contact information, an ingredient list and the recommended daily dose [5]. As food supplements are not always sold from Danish stores but are instead purchased online from companies registered within but also outside of the EU, some food supplements do not necessarily comply with EU or national regulations.

Food supplement use is high in the general Danish population compared with other European countries [6,7]. It is a common finding in surveys from both Europe and the US that food supplement intake increases with age and education level, and is more popular among women compared with men [6,8]. However, the few studies that exist on male recreational athletes training in gyms indicate that men may have a higher intake of food supplements and protein products compared with women [9,10].

Protein is a popular ingredient in products marketed to athletes. The World Health Organization (WHO), Food and Agriculture Organization and United Nations University have defined the recommended daily allowance for good-quality protein to be 0.83 g/kg body weight for healthy adults at all ages. For the general population, a varied diet that meets energy requirements is likely to supply more than adequate amounts of protein [11].

Caffeine (1,3,7-trimethylxanthine, CAS no 58-08-2) is a naturally occurring substance found in, e.g., seeds from coffee (*Coffea arabica* L.), cola (*Cola nitida* (Vent.) Schott & Endl.), guarana (*Paullinia cupana* Kunth) and in leaves from the tea bush (*Camellia sinensis* (L.) Kuntze) and maté (*Ilex paraguariensis* A.St.-Hil.). Coffee and black tea have been popular hot beverages for hundreds of years in Europe due to their taste and stimulating properties. Later caffeine-containing soft drinks, e.g., cola, were introduced in Europe and, within the last two decades, energy drinks as well. Other sources of caffeine are food supplements and pre-workout (PWO) products. PWO products are not defined in any regulation but are marketed for use before training. They are characterized by a content of multiple ingredients purported to increase energy, extend endurance and boost muscle gain. Evidence to support the performance-enhancing activity of the blend of ingredients is often scarce or lacking [12] and some products have added stimulant substances and/or ingredients of unknown safety [12,13].

Caffeine is regulated as a flavoring substance and may be added to non-alcoholic beverages such as cola at a level of up to 150 mg/kg [14]. However, energy drinks may contain caffeine levels higher than 150 mg/kg and up to 320 mg/kg if labeled with the mandatory warning: ‘High caffeine content. Not recommended for children or pregnant or breast-feeding women’ [15]. Energy drinks that most often, apart from caffeine, contain taurine and D-glucurono-γ-lactone, among other ingredients, are promoted for their actual or perceived effects as stimulants, energizers and performance-enhancers [16,17,18], and their marketing is primarily targeted toward young men [18,19]. Marketing of energy drinks with a higher caffeine content than caffeinated soft drinks became legal with a restricted unit size of 25 cl after a change in Danish regulation in November 2009. In the summer of 2011, the unit size restriction was lifted [15]. After these changes, the amount of energy drinks sold in Denmark has increased more than fourfold, from approximately 6.3 million liters annually in 2010 to more than 26 million liters in 2018, and with an expected further increase to 34.6 million liters in 2023 [20].

In recent years, the intake of green tea infusion has increased in popularity in Europe, owing to real or perceived health benefits [21]. Additionally, green tea extracts, often standardized to high levels of specific catechins, especially (-)-epigallocatechin-3-gallate (EGCG), have become one of the largest ingredient segments for food supplements on the global market [22]. Among consumers, there is a general belief that green tea or green tea extract has a weight-reducing effect [23]. However, the scientific evidence is lacking [24,25].

It is common that male professional athletes ingest food supplements or extra protein as observed in, e.g., British and German elite athletes [26,27]. It is a finding, at least from the US, that elite athletes, uniformed military service members and young active adults use higher amounts of supplements than the general population. These subpopulations are therefore at greater risk of experiencing adverse effects after intake of such products [28,29]. Generally, the intake of food supplements and protein supplementation among professional or collegiate athletes has gained some research interest [26,27,30,31,32]. The intake of these products in young men aiming to increase their muscle mass by training in commercial gyms has only been surveyed in few studies [9,33,34,35,36]. Danish intake data for these products in this subgroup of the population do not exist. Caffeine-containing beverages such as coffee, tea, caffeinated soft drinks and energy drinks may be consumed as part of the common lifestyle or for their stimulating properties when training. Additionally, food supplements containing caffeine may be ingested to increase training performance in recreational athletes. No studies have so far looked into caffeine intake from coffee, tea, energy drinks and food supplements in young men exercising in gyms.

The present study aimed to investigate the use of food supplements, protein products and caffeine- and green-tea-containing products among young Danish men attending one of the most popular chains of commercial gyms to increase muscle mass, and to investigate the intake of protein, caffeine and EGCG. The participants were also questioned about the reason for using the products, whether they had ever experienced adverse effects after intake and, if so, whether they had consulted a physician or reported the adverse effects to the DVFA.

## 2. Materials and Methods

### 2.1. Selection of Gyms

A pilot study was performed with the purpose of identifying gyms that were attended by the target group: recreational male athletes between 15–35 years of age exerting a muscle-building workout. Three different gym chains located in the Copenhagen area were visited. One of the chains was most popular among the target group and was chosen for the recruitment of study participants.

### 2.2. Subjects and Study Design 

Study participants were recruited from six different gyms from the chosen gym chain in the Copenhagen area from February to April 2016. All young men entering the gyms between midday and 6 p.m. on a random weekday were invited to participate in the study. Inclusion criteria were men aged 15–35 years, performing muscle-building workouts and taking food supplements. In total, 72 subjects accepted the invitation for an interview and 63 completed the study. The reason for the opt out of nine participants was insufficient knowledge about their ingested products. All interviews were anonymous, face-to-face, based on a questionnaire and conducted by the same investigator. Completion of the interview was considered as consent to participate in the study. The present study adheres to the General Data Protection Regulation. The Danish National Committee on Health Research Ethics has decided that, according to Danish Law, the study does not require their approval.

### 2.3. Questionnaire

A questionnaire was developed to provide information on the intake of food supplements and protein products, brand names, intake of caffeine and green tea from all dietary sources, and protein from ingested products. The questionnaire included questions on (1) age, (2) practice of other sports, (3) training quantity at the gym (including hours per week spent on muscle-building workouts), (4) use of food supplements and protein products (brand names, dose used and frequency), (5) intake of dietary sources of caffeine such as coffee, tea (black, white and green), caffeinated soft drinks and energy drinks as well as caffeine-containing products (brand names, intake and frequency), (6) reason for use of supplement or protein products (including whether the products were used due to specific ingredients), (7) sources of supplement/protein product information, (8) awareness of the notification of the food supplement in question to the DVFA, and warnings regarding the specific product, (9) whether the participant had ever experienced adverse effects after intake of food supplements or other products and (10) whether a physician was consulted in case of experienced adverse effects. The acceptability of the questions was tested before use. To test whether the questions were understandable, the first draft of the questionnaire was initially tested with five volunteers. Secondly, the data collection was tested in the pilot study including six different gyms from three gym chains before deciding on the final wording in the questionnaire.

### 2.4. Data Analysis

The notification status of all used food supplements was checked in the DVFA database [37]. Information on the contents of protein, caffeine and green tea or EGCG was obtained by reading the product label, by consultation of the manufacturers’ homepages and in some cases by contacting the manufacturer/provider by e-mail or phone. If data were still not provided, a mean caffeine content of 9.2 mg caffeine per 100 mL of caffeinated soft drink was assumed, corresponding to several average measurements of the caffeine content in carbonated soft drinks [38]. For an estimation of caffeine intake from other hot and cold beverages the following average contents of caffeine per 100 mL of ready-to-drink product was used: coffee: 50 mg, espresso: 67 mg, energy drinks: 15–32 mg (as labeled ), black tea: 18.5 mg and green and white tea: 11.5 mg per 100 mL [16,39]. The information was used for calculating the individual caffeine intake from all sources. For estimation of the EGCG intake from green tea, a mean content of 0.7 mg per g of brewed tea was assumed [40].

Data obtained from the completed interviews were used to create a product database containing information on identified supplements (name, ingredients and amount of substance per dose). The database was used for extracting information for calculation on the mean daily intake of protein, caffeine and EGCG from food supplements and protein products for the three age groups: 15–17, 18–24 and 25–35 years of age. Since we did not collect information on individual body weights, to estimate the caffeine intake per kg body weight, average body weights for 15-, 16- and 17-year-old Danish male adolescents (67.1, 68.8 and 80.8 kg, respectively) were obtained from the Danish National Survey on Dietary Habits and Physical Activity (DANSDA) from 2011–2013 [41]. Data on the protein intake from food were not collected as part of the study. Therefore, data for the three studied age groups were from DANSDA. For the calculation of the protein intake per kg body weight, the average body weights of Danish men aged 15–17, 18–24 and 25–35 years (71.1 kg, 78.4 kg and 85.7 kg, respectively) were obtained from DANSDA [41].

## 3. Results

### 3.1. Subjects and Training Characteristics

In total, 63 males (aged 23.2 ± 5.1 years (mean ± SD), range 15–35) completed the interview. Participants aged 15–17, 18–24 and 25–35 years attended 2–6, 2–7 and 2–6 training sessions per week, respectively (Table 1). Each training session lasted 60–150, 30–150 and 60–150 min in the three age groups of which 30–150, 30–120 and 30–150 min, respectively, were spent on muscle building. The number of training sessions per week was higher among the oldest age groups but training duration per session was similar among the groups. Furthermore, 29% of the 18–24-year-old men were involved in other sport activities, and among the 15–17 and 25–35-year-old participants this number was slightly higher (37.5%).

### 3.2. Supplement Use

One hundred and thirty-six different food supplements and protein products were recorded (Table 2). Seventy-four of these products (54%) were labeled as food supplements. Only 32 (43%) of these were notified to the DVFA meaning that they were legally registered for sale in Denmark. The most common products used by the participants were protein powder (60%), fish oil (54%) and multivitamin/mineral supplements (41%). In addition to protein powder, other sorts of protein products were common, including bars (25%), shakes (16%) and specific branched-chain amino acid products (21%). Most of the participants were not able to recall the brand names of the various vitamin and mineral supplements they reported taking and therefore the vitamin and mineral intake from the food supplements were not quantifiable.

### 3.3. Reason for Use of Food Supplements and Protein Products and Sources of Product Information

The main reason for using food supplements and protein products was fitness related, such as maintenance (16%), muscle building (57%) and performance enhancing (17%). Other participants regarded them as part of a healthy lifestyle and necessary for staying healthy and physically active (30%), while some took protein products to ensure an adequate protein intake (10%).

Thirty-four participants (54%) read about the products on the internet, and thirty (48%) were introduced to them through friends and acquaintances. Additionally, 16 participants (25%) mentioned other sources of information such as television commercials (3%), their physician (8%), their fitness instructor (2%) or access due to their all-in subscription at the gym (3%).

### 3.4. Protein Intake

Fifty-two out of the sixty-three participants (83%) consumed protein products on a weekly basis with protein powders being the most popular product used by 60% (Table 2). Intake of protein from different protein products increased with age (Table 3). All eight 15–17-year-olds reported taking protein-containing products, while the proportion of users was slightly lower among the elder age groups (between 74% and 88%). Supplementary protein intake among the 18–35-year-old consumers with the highest intake (113 g protein per day, reported by two participants) was more than four times the highest reported intake among the 15–17-year-olds and corresponds to 1.44 and 1.32 g/kg body weight per day for 18–24- and 25–35-year-old men, respectively (Table 3). Six of the adult protein supplement users (14%) exceeded the recommended daily allowance for protein (0.83 g/kg body weight) with their intake of supplementary protein. Fifteen of the adult protein supplement users (34%) had a total estimated protein intake exceeding 1.66 g/kg body weight per day (twice the recommended daily allowance).

### 3.5. Caffeine Intake

**Figure 1:**
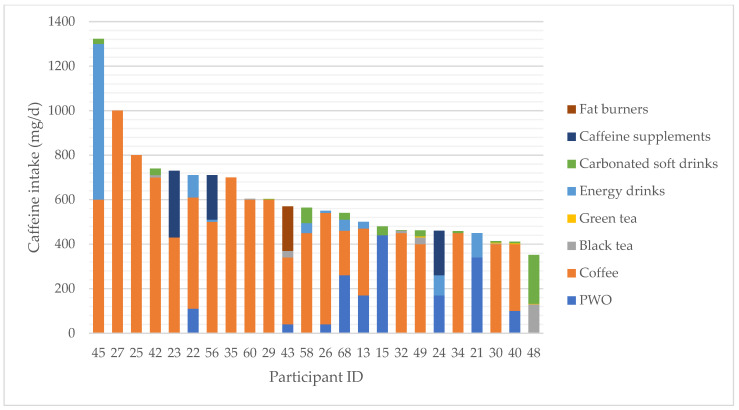
Individual caffeine intake by source for participants exceeding the presumed safe level (for caffeine, an intake up to 400 mg per day from all sources does not give rise to safety concern in adults). For children and adolescents, an intake of 3 mg/kg body weight per day may serve as a basis to derive intake of no concern for this subgroup [16]. The last bar is showing intake from an adolescent; the other bars represent intake data for adult men (18–35 years). PWO: Pre-workout products.

All participants had a caffeine intake varying from 5–1323 mg per day. Thirty-eight of the participants, corresponding to 60%, drank coffee of which 66% drank it daily. Other sources of caffeine were black and green tea, PWO products, fat burners, energy drinks and carbonated soft drinks. Twenty-three adult males ingested more than 400 mg caffeine per day and one adolescent exceeded the presumed safe level for his age group (Figure 1). In total, 21 respondents (33%) reported an energy drink intake. One participant had a daily intake of 700 mg caffeine from energy drinks. Eight (13%) participants drank some kind of caffeinated soft drink or ingested PWO products before or during their workout. Due to the lack of information on the caffeine contents in some of the supplements, the intake of caffeine from these products could not be included in the intake estimates.

### 3.6. Green Tea and EGCG Intake

Nineteen (30%) participants reported drinking green tea. Two of the participants had an intake of 1.5 and 2 liters per day, corresponding to a daily intake of 1050 and 1400 mg EGCG, respectively. The other 17 green tea drinkers reported an intake of between 100–600 mL per day. Some food supplements such as fat burners contained green tea extracts. However, no information on the actual contents of EGCG was labeled in the list of ingredients. Therefore, the intake of EGCG from supplements could not be calculated.

### 3.7. Adverse Effects

Eighteen (29%) participants had experienced adverse effects, and fifteen of them recalled the brand name or the product category (Table 4). The last three participants had experienced adverse effects (unspecific symptoms from the gastrointestinal tract or tingling) but were unable to remember which product caused the effect and data from these are therefore not included in the table. Most adverse effects (53%) were associated with the intake of PWO products, where users reported insomnia, shaking, headache, palpitations, itching of the skin and stinging after intake.

One participant consulted a physician after experiencing an adverse effect, indigestion, after intake of protein shakes. He was diagnosed with lactose intolerance.

### 3.8. Product Notification and Safety Alerts

Fifty-eight participants (92%) were not aware of whether the consumed food supplements were notified to the DVFA and legally sold in Denmark. Three (5%) reported that all of their products were notified and two (3%) reported that some of the products were notified. Among the participants, 20 (32%) followed the safety warnings from the DVFA concerning unsafe brands of food supplements, 39 (62%) participants were not aware that such warnings existed and 4 (6%) participants were aware but did not follow them.

## 4. Discussion

Important findings in our study are that intake of supplemental protein, coffee and other caffeine sources are popular among young Danish men training to increase muscle mass in gyms and that it is not uncommon that they have experienced symptoms of adverse effects after intake of food supplements, especially PWO products.

### 4.1. Training

In the present study, all participants trained at least two to three times every week, and the older age groups trained more frequently than the youngest one. On the other hand, the youngest men were the ones spending the most time on muscle-building activities. These results are in line with a finding from Brazil where the majority (85%) of male and female gym goers trained at least three times a week, and 75.3% spent more than one hour a day exercising [9]. 

### 4.2. Supplement Use 

In Denmark, use of food supplements is common [42]. It is, however, not possible to estimate the percentage of food supplement users among male recreational athletes from our data since only food supplement users were recruited. In the only other Danish study from 2014 on male recreational athletes (*n* = 265, age: 15–49 years) training in commercial gyms and almost exclusively performing strength training, nutritional supplement use was reported in 94% [43]. In comparison, food supplement use was 50.8% in the general population of Danish men in the age group 18–25 years and 54.9% in men aged 26–35 years [42]. The study by Solheim et al. [43] uses a definition of nutritional supplements including products containing ingredients such as vitamins, minerals, fatty acids, caffeine, beta-alanine, bicarbonate and creatine, but also products such as protein powders and sport drinks as well as food supplements such as PWO products, ‘fat burners’ and ‘energy pills’. Further investigation is needed to clarify whether the very high supplement use is representative of Danish male recreational athletes, since the men in the study were targeted for doping control and the definition of nutritional supplements was broader than the legal definition of food supplements.

In our study, the most frequently used supplements were protein-based products (83%) followed by fish oil (54%) and multivitamin/mineral supplements (41%). No other Danish studies were identified in the literature examining protein and food supplement intake in young recreational athletic men. Multivitamin/mineral supplements (43%) and fish oil (18%) are the most common food supplements taken in Denmark by adult men (18–75 years) in the general population according to data from DANSDA from 2011–2013 [42]. Studies from other countries have also shown that people who exercise in gyms are frequent users of protein products and food supplements but that national preferences prevail. Our study is in line with a recent Portuguese study finding that protein, creatine and branched-chain amino acids were the most popular supplements among young men attending gyms to exercise [10]. In a Brazilian study, the most commonly used supplements among men were products rich in protein, products rich in carbohydrates and isotonic drinks [9]. Another survey performed in the US, including primarily men (82.0%), reported that among the 84.7% who took supplements, 54.9% consumed protein shakes and bars, 33.3% consumed creatine, 30.2% consumed carbohydrate shakes/bars and 45% consumed multivitamin/minerals on a regular basis [34]. A South African study found that after protein products, energy drinks were the most popular among men exercising in gyms [44]. A preference for the intake of supplemental protein or amino acids seems to be a general finding in studies on supplement use among young men exercising in gyms, irrespective of the nation in which the data were collected. However, the present study also elucidated national preferences. Food supplements such as multivitamin/mineral tablets and fish oil capsules are popular in the general Danish population and were also commonly used by the study participants. Whereas the intake of supplemental multivitamins/minerals was also seen in some other studies, the preference for intake of fish oil was specific to the men in our study.

Twenty-one percent of the men ingested PWO products. Little information on the use of PWO products among recreational athletes exists, but this percentage is higher than German data collected in 2016 finding that 11.8% of food supplement users (both men and women) attending gyms had used PWO products within the last month [13]. However, PWO products seem to be popular among active-duty soldiers in the Australian army where 28% of supplement users reported intake of PWO products [45].

### 4.3. Reasons for the Use of Food Supplements and Protein Products and Sources of Product Information

The primary reasons for taking food supplements were to build muscles (57%) and to enhance performance. Other participants took them as part of what they perceived as a healthy lifestyle and some listed restoring nutrients/avoiding weakness as the main reason. The focus on muscle mass was in line with two studies of younger (up to 30 years of age) gym goers (both men and women) from Brazil and the US where 47.0 and 56.3%, respectively, of food supplement users reported that they took supplements to increase muscle mass [9,34].

Half of the men in the present study used protein or food supplements after having read about the products on the internet and the other half had them recommended by friends. A study from the US from 2004 found that the most common sources of information on supplements were magazines (65.8%) and family/friends (63.1%) but personal trainers (38.7%) also played a role [34]. It seems likely that easy access to the internet has changed the main sources of information about the products from magazines to the internet. These findings differ from a study performed in the UK, Turkey and Italy, where the coach in 46–65% of cases was the person recommending protein supplement use [46]. In Denmark it is uncommon for recreational athletes to have a personal coach, so it is not surprising that coaches are playing a minor role in recommending an intake of supplements.

### 4.4. Protein Intake

Estimated protein intake from protein-containing supplements increased with age. Assuming a recommended daily protein allowance of 0.83 g/kg body weight for adults [11], the data show that six adult participants with the highest protein intake from supplemental protein products had an adequate intake from the protein supplements alone. In Denmark, the estimated average dietary protein intake from DANSDA (for the three investigated male age groups) corresponds to 1.21–1.36 g/kg body weight per day [41], showing that the protein recommendations are fulfilled by diet alone in the general Danish population. Assuming a dietary protein intake corresponding to the intake among peers, the total estimated protein intake (from the habitual diet and supplemental protein) for 34% of the adult protein supplement users exceeded the daily intake level of 1.66 g/kg body weight (twice the recommended intake) that according to WHO [11] is considered unlikely to be associated with any risk. The highest intake equals 3.1 or 3.3 times the recommended intake. The EFSA (European Food Safety Authority) and WHO found that data were insufficient to establish a safe upper limit for protein intake [11,47]. According to WHO [11], a very high intake of 3–4 times the recommended daily allowance approaches the tolerable upper intake limit and cannot be assumed to be risk-free whereas the EFSA [47] stated that an intake of 3–4 times the recommended daily allowance has been observed without apparent adverse effects. Whether the total estimated protein intake of the high consumers of protein food supplements in the present study is risk-free therefore needs further investigation. 

The majority of training was spent on muscle-building activities, which could explain the high use of protein products. 

### 4.5. Caffeine Intake 

According to the EFSA [16], a total daily intake of 400 mg of caffeine does not give rise to safety concern in the general, healthy, adult population. For children and adolescents, the available information was insufficient to derive a safe level of intake but the EFSA panel suggested that an intake of 3 mg/kg body weight per day may serve to derive intake of no concern for this subgroup. The EFSA also noted that 100 mg of caffeine may increase sleep latency and reduce sleep duration in some susceptible individuals, particularly when consumed close to bedtime [16]. According to Nawrot et al. [48], habitual daily use of caffeine in doses exceeding 500–600 mg may lead to adverse reactions such as restlessness, anxiety, irritability, agitation, muscle tremor, insomnia, headache, diuresis, sensory disturbances (e.g., tinnitus), cardiovascular symptoms (e.g., tachycardia, arrhythmia) and gastrointestinal complaints (e.g., nausea, vomiting, diarrhea). 

Coffee was the primary source of caffeine in the present study. The average intake of coffee among Danish men aged 15–24 and 25–44 years is 0.6 and 3.7 cups per day, respectively [49]. Sixteen (25%) of the men exceeded the level of caffeine of 400 mg per day by drinking coffee. Twenty-three (37%) of the sixty-three participants over 18 years surpassed the 400 mg of caffeine intake per day. One participant under 18 years possibly surpassed the upper level for caffeine intake per day [16] considered to be without safety concern, when we assume a body weight of 68.8 kg (corresponding to the average body weight of Danish male teenagers of 16 years of age according to DANSDA) [41]. Even though the caffeine intake was high, it may still be underestimated due to the lack of information on caffeine content in some of the food supplements. Food supplements on the Danish market should be labeled with the caffeine content if the substance is added as such or if the label mentions caffeine as a characteristic substance of a plant extract used, but not if a plant extract is labeled on the list of ingredients without further mention of caffeine [50].

According to our data, a higher proportion (33%) of men exercising in gyms use energy drinks compared with similar age groups of the general population. Among Danish men with a high habitual intake of energy drinks (at least 4 times a week), 7% of the adolescents (15–19 years), 9% in the age group from 20–26 years and 11% in the group of men aged from 27–35 years had a high intake [51]. Our findings are in line with a Canadian study showing that advertising for energy drinks is perceived as targeting young people and promoting use during sports [19]. In the present study, the intake of energy drinks may pose a risk since one participant had an intake of caffeine of 700 mg per day from energy drinks alone.

### 4.6. Green Tea and EGCG Intake 

In this study, most of the men drinking green tea had a more moderate intake, but the excessive intake reported by two of the men was an unexpected finding. They drank 1.5 and 2 L green tea daily, exceeding not only the 95 percentile of tea intake for Danish adult men of 1171 g per day but also the 97.5 percentile of 1422 g per day for men regularly drinking all sorts of tea [41]. Intake of 1.5 and 2 L green tea corresponds to a daily intake of 1050 or 1400 mg EGCG, respectively. In the EU, the mean daily intake of EGCG resulting from the consumption of green tea infusions ranged from 90 to 300 mg per day while exposure by high-level consumers was estimated to be up to 866 mg EGCG per day in the adult population [40]. According to the EFSA, green tea infusion prepared in a traditional way is in general considered safe provided the intake corresponds to reported intake in European Member States. However, rare cases of liver injury have been reported after consumption of green tea infusions, most probably due to an idiosyncratic reaction [40]. Cases of hepatotoxicity after intake of green tea infusion have been reported after daily intake of 2–6 cups for 3–9 months [52,53,54]. Even though only a few cases of hepatotoxicity have been described after drinking green tea, the safety of such very high consumption is not well established.

Green tea as a beverage is not the only source of EGCG since green tea extracts are also ingredients in food supplements. However, information on the quantity of green tea extracts or EGCG in the supplements were lacking, making it impossible to estimate the actual intake of EGCG from this source. Furthermore, analytical data on the contents of total catechins and EGCG may vary considerably. A study on food supplements from the US found that the contents of green tea material does not permit accurate predictions for the content of specific phytochemical constituents. For example, at the most commonly labeled level for green tea material (500 mg/serving, *n* = 9), the analytical mean values for total catechins ranged from 1.4–410.6 mg/serving, and from 0.5–314.8 mg/serving for EGCG. Of 18 food supplements, for which the companies had added voluntary label information on EGCG contents, percent differences from the label ranged from 35% below the label to 186% above the label, with 10 products within ±20% of labeled contents [55]. 

Based on the available data on the potential adverse effects of green tea catechins on the liver, the EFSA [40] concluded, based on evidence from interventional clinical trials, that intake of doses equal to or above 800 mg EGCG per day taken as a food supplement may induce a statistically significant increase in serum transaminases in treated subjects compared with control subjects. Two papers have suggested an upper tolerable intake level of 300 mg EGCG per day for food supplements containing green tea extracts [56,57].

The EFSA recommends that labels of food supplements should include information on the content of catechins as well as the proportion of EGCG [40] but this recommendation has so far not resulted in an EU regulation. Outside of Europe, The United States Pharmacopeia’s ongoing review of dietary supplement safety data has recently included a cautionary labeling requirement in its monograph on powdered decaffeinated green tea extract saying, ‘Do not take on an empty stomach. Take with food. Do not use if you have a liver problem and discontinue use and consult a healthcare practitioner if you develop symptoms of liver trouble, such as abdominal pain, dark urine, or jaundice (yellowing of the skin or eyes)’ [58].

### 4.7. Adverse Effects 

PWO products were linked to various acute effects such as shaking, sweating and flickering of the eyes, palpitations, red skin or stinging, itching and headache, or later insomnia. Some PWO products may contain substances with unknown risks and are included among the higher-risk supplements by some authors [29,59]. Our study supports the notion of PWO products being of higher risk than other supplements since more than half of the reported adverse effects were after intake of supplements classified as PWO products.

The reported adverse effects are remarkable but only one participant consulted a physician following another kind of side effect, indigestion, after drinking protein shakes and was subsequently diagnosed with lactose intolerance. None of the participants reported their adverse effects to the DVFA. The DVFA has on its homepage a form where consumers may report adverse effects following intake of food supplements [60]. So even though a Danish nutrivigilance system exists for reporting adverse events caused by food supplements [61], the young men for some reason did not report the adverse effect they experienced. As found in other studies, adverse effects of food supplements are often underreported [62,63].

The percentage of men experiencing adverse effects (29%) is higher compared with data from the only other published study on recreational athletes exercising in gyms. In this Brazilian study, 5.5% of the participants (both men and women) reported adverse effects after the ingestion of food supplements [9]. Some of the effects were alike, e.g., dizziness, impact on the skin and insomnia, whereas others, e.g., impact on the liver or kidneys were not reported by the Danish men. It is remarkable that so many recreational athletes had experienced some adverse effect after supplement intake. In comparison, 16% of regular food supplement users among active-duty soldiers in the Australian army reported adverse effects [45].

Common features of caffeine intoxication include anxiety, restlessness, insomnia, gastrointestinal upset, muscle tremor and tachycardia [48] and, in rare cases, death [64,65,66]. The symptoms of caffeine intoxication can mimic those of anxiety and other mood disorders [67], and the reported adverse effects could potentially be linked to the caffeine content of the products in combination with a high caffeine intake from coffee. However, some of the effects may also be linked to other labeled or unlabeled substances in the supplements.

Three of the participants reporting adverse effects had used food supplements for which the DVFA had previously issued warnings to the public. One of the men experienced insomnia after intake of the product Craze produced in the US and sold on the internet. Intake of this food supplement for two weeks had in 2013 given rise to the hospitalization of a young Swedish man with tachycardia. According to the label, the ingredients in Craze were caffeine and an extract of the fruit of *Citrus reticulata* and a *Dendrobium*-extract containing, among others, substances of a mixture of phenylethylamines. Phenylethylamine derivatives are not naturally present in species belonging to the genus *Dendrobium* [68]. N,α-diethyl-phenylethylamine, a structural analogue of methamphetamine, was identified in three separate lots of Craze [69] and a Swedish study also found, apart from N,α-diethyl-phenylethylamine, N,β-diethyl-phenylethylamine and N,N-diethyl-phenylethylamine [70]. The safety of the three phenylethylamines in humans are entirely unknown [69]. The US Food and Drug Administration (FDA) sent a warning letter to the manufacturer of Craze in April 2014 considering the product as adulterated, but it is not clear whether the product was removed from the market [71]. Phenethylamine (synonym phenylethylamine) and its derivatives are listed as stimulants by the World Anti-doping Agency (WADA) and prohibited substances in competition [72] meaning that if any of the participants were professional athletes they ran the risk not only of adverse effects but also of testing positive for doping and being suspended from their sports. Since the majority of Danish commercial gyms are subject to doping control, the recreational athletes may also be selected for doping control [43].

Two of the participants experienced shaking or headache after ingestion of the PWO product Jack3D. This is another popular supplement manufactured in the US and accessible worldwide through sales online. The supplement contained 1,3-dimethylamylamine (DMAA), claiming it was an inherent substance in some plants. However, DMAA was not of natural origin but an added obsolete drug (decongestant) [73,74,75,76]. DMAA had never been registered as a medicinal agent for oral use, but only for nasal application where a dose of about 0.6 mg was considered therapeutically active. Very limited toxicological data exist on its safety [77]. The DMAA content was not declared on the label of Jack3D but various studies have reported a content per serving size of 27, 36 or 142 mg [74,75,78] meaning that consumers were exposed to a much higher intake than if administered as a drug. Intake of DMAA has been linked to a considerable number of serious adverse effects and even deaths [79,80,81,82]. An Australian Poison Information Center reported a diverse list of symptoms after intake of DMAA with the most common being nausea and vomiting, palpitations and tachycardia, headache, anxiety and agitation but also chest pain, tremors and shakiness [83]. The headache and shaking described by the two men after intake of Jack3D in our study are therefore similar to the adverse effects reported by others.

DMAA (under the synonyms 4-methylhexan-2-amine and methylhexaneamine) was in 2010 added to the list of stimulants prohibited in competition by WADA [84]. In Denmark, the DVFA first warned against the use of a food supplement containing DMAA in November 2011 and several times thereafter. In 2013, the US FDA warned manufacturers and companies marketing food supplements with DMAA that their products contained an illegal ingredient and warned consumers against their use [85]. Efficiently removing dangerous supplements from a transnational market seems to be difficult [86]. In a study from the US, 66.7% of food supplements recalled by the US FDA for being adulterated or containing banned ingredients were still available for purchase in the US at least 6 months after FDA recalls [87]. Years after DMAA was considered dangerous both in the US and Europe, it was still found in some supplements on the US market in 2016, and in the same year the EU Rapid Alert System for Food and Feed received 16 notifications of findings of DMAA in products marketed in EU member states [88,89]. In May 2022, the DVFA again issued a warning to the public after having found a food supplement labeled with a content of DMAA marketed on a Danish website. 

Another ingredient in food supplements targeted at athletes is beta-alanine, a non-essential amino acid, produced endogenously and found in our food. In Denmark, food supplements may be added up to 1 g beta-alanine per the recommended daily dose [90]. The estimated daily dietary intake of beta-alanine from food is 3–4 g in the general population. Beta-alanine is a popular amino acid supplement and has been linked to increased physical performance and endurance in studies with an intake of 4 g per day or more for shorter periods of time [91]. However, the scientific evidence was not considered strong enough for providing a health claim for beta-alanine when evaluated by the EFSA [92]. One participant experienced tingling of the skin (paraesthesia) after ingesting beta-alanine supplementation. The only known adverse effect after beta-alanine supplementation is paresthesia, which dissipates within one hour after ingestion and is usually associated with ingestion of more than 800 mg of beta-alanine (10 mg/kg body weight) [91,93]. Similar amounts of beta-alanine ingested from foods do not give rise to adverse effects. Even though the reported symptom resembles an adverse effect caused by intake of too high doses of beta-alanine, the participant was not able to recall the dose he ingested and a cause and effect relationship cannot be established with certainty.

Intake of a so-called weight gainer resulted in chest pain in one participant. Chest pain is not necessarily a harmless symptom but since no further information on the brand name of the supplement or its constituents is available, it is impossible to deduce whether the product may have contained unsafe ingredients or caused the effect.

Buying supplements from the internet may have become popular as described in a recent Portuguese study where 68% of male gym goers bought their food supplements through the internet with supplement/health food stores being the second place of choice [10]. The participants in the present study were not asked where they purchased their supplements but since only 32 of the 55% of products labeled as food supplements were notified to the DVFA, it may be a qualified guess that a substantial amount were bought outside of Denmark. Considering the difficulties in the removal of dangerous products nationally and internationally, the easy access through online sales, the consumer perception of food supplements as safe products and the low perception of symptoms of an adverse effect may be a dangerous cocktail. Due to the international nature of the sales of food supplements, there is a need for better education of the consumers and a need for increasing their awareness of unsafe products. A readily accessible resource is the Operation Supplement Safety website (OPSS.org) developed in the US as an educational campaign to increase awareness within the military community about how to make informed decisions on food supplements and avoid potential health risks [94]. A similar database accessible to the general European consumer could also be highly relevant.

### 4.8. Product Notification and Safety Alerts 

The majority of the recreational athletes in our study had not sought professional advice before using protein supplementation or food supplements but had read about the products on the internet or had friends or acquaintances introducing them to the products. Even when experiencing adverse effects such as headache, shaking, palpitations, stinging or other less innocuous symptoms they did not seek medical advice. This is in line with a finding of a low perception of risk among gym goers using food supplements. In the same study, the fitness trainers believed that the benefits of ingesting supplements exceeded the risk whereas the view of interviewed dieticians was that taking supplements involved risks and professionals with proper nutritional knowledge should guide supplement use [95]. 

### 4.9. Strengths and Limitations 

One major strength of the present study is the use of a questionnaire administered by the same investigator. Using face-to-face interviews to collect data reduces bias from a self-administered questionnaire [96] which is the preferred method in other similar investigations in other countries. The difference in data collected by a written questionnaire compared with a structured interview was highlighted by Henserud and co-workers finding that the prevalence of reported use of dietary supplements was 30.5% when answering in writing in comparison with 61.0% reported during the structured interview [97]. Jeurissen and co-workers conducted a survey of plant food supplement use in the Netherlands based on an online questionnaire and described that the responders had difficulties in distinguishing food supplements from traditional or regular herbal medicinal products and homeopathic products [98]. Another strength of our study was that the interviewer was present and able to classify the product used together with the study participants on the day of the interview. 

Due to the study design where the interviewer recruited study participants on a random day at the selected gyms, we cannot exclude that the study participants are among those who train the most, since the probability of recruiting men who train less often is lower when recruitment only happens on one day per gym. We can therefore not exclude that the training segment recruited is more focused on performance and muscle building than would be the case had we chosen to recruit at each gym on more weekdays.

Another limitation of the present study is that only supplement use was recorded. No dietary records were collected. The estimated dietary protein intake obtained from DANSDA [41] was used in the dietary intake calculations and may not be representative of the investigated group. A study found that supplement users among recreational athletes had higher protein intake from their diet compared with non-supplement users [35]. Therefore, we might have underestimated the protein intake from their diets that could in reality be even higher. On the other hand, since we did not collect data on habitual protein intake we might have overestimated dietary intake. A lack of individual body weight data also affects the calculated maximum intake level for caffeine in subjects below the age of 18 years. We used the mean body weights of adolescents of a similar age extracted from DANSDA [41].

## 5. Conclusions

Protein products were the most popular supplements among young male recreational athletes training in gyms for gaining larger muscle mass. The total estimated protein intake from supplements and diet reached 3.1 or 3.3 times the recommended daily allowance for protein for two participants. The intake of other products was accompanied by a number of potential safety concerns: high intake of caffeine, a few participants having an extreme intake of green tea and a substantial number of the participants having experienced adverse effects after intake of food supplements. The latter had neither been discussed with their physician, nor been reported to the food authorities.

## Figures and Tables

**Table 1:** Training characteristics (number of training sessions per week, time spent on each training session, specific muscle-building training and involvement in other sport activities) reported by the 15–35-year-old men participating in the survey.

Age, Years	15–17	18–24	25–35
*n*	8	31	24
Training sessions per week, *n* (%)			
2–3 times	3 (37.5)	4 (13)	2 (8.5)
3–4 times	2 (25)	2 (6)	2 (8.5)
4–6 times	3 (37.5)	20 (65)	20 (83)
7 times	0	5 (16)	0
Training time per session, *n* (%)			
30–60 min	0	6 (19.5)	0
60–90 min	3 (37.5)	14 (45)	15 (62.5)
90–120 min	4 (50)	10 (32.5)	6 (25)
120–150 min	1 (12.5)	1 (3)	3 (12.5)
Muscle-building training per session, *n* (%)			
30–60 min	1 (12.5)	11 (35.5)	8 (33.5)
60–90 min	6 (75)	13 (42)	12 (50)
90–120 min	0	7 (22.5)	3 (12.5)
120–150 min	1 (12.5)	0	1 (4)
^1^ Involved in other sport activity, *n* (%)	3 (37.5)	9 (29)	9 (37.5)

^1^ Sport activities included football, American football, cycling, running, cross fit, softball and badminton.

**Table 2:** Users of food supplements and protein products among men attending gyms to increase muscle mass, number of different products used within each category and information regarding labeling and notification to the Danish Veterinary and Food Administration.

	Users (%)	Products (*n*)	Labeled as Food Supplement (*n*)	Not Notified (*n*)
Protein powder	60	32	2	2
Protein bar	25	12	0	0
Protein shake	16	7	0	0
Weight gainer	6	6	4	4
BCAA	21	10	9	7
Creatine	13	12	4	4
PWO product	21	10	10	8
Fat burner	2	1	1	1
Ginseng	2	1	1	1
Ginger	2	1	1	0
Glucose	2	2	0	0
Fish oil	54	18	18	6
Multivitamin	41	9	9	4
Vitamin D	16	6	6	3
Vitamin D + calcium	2	2	2	0
Vitamin B	2	1	1	0
Zinc	2	1	1	0
Magnesium	3	2	2	1
Caffeine	8	3	3	1
Total		136	74	42

Abbreviations: BCAA: branched-chain amino acids. PWO: pre-workout.

**Table 3:** Estimated mean protein intake from protein products in 15–35-year-old men attending gyms to increase muscle mass. Intake is calculated assuming average body weights (71.1, 78.4 and 85.7 kg for 15–17-, 18–24- and 25–35-year-old Danish men, respectively) obtained from The Danish National Survey on Diet and Physical Activity [41]. The mean estimated supplementary daily protein intake results are from our study, whereas the mean estimated dietary protein intake in the general Danish male population is calculated based on data from [41].

Age (Years)	15–17	18–24	25–35
*n* (% users)	8 (100%)	31 (74%)	24 (88%)
Mean estimated supplementary protein intake * (g per day)	16.3 ± 8.3 (5.7–26.6)	24.7 ± 27.5 (0–113)	31.2 ± 31.4 (0–113)
Users only: mean estimated supplementary protein intake * (g per day)	16.3 ± 8.3 (5.7–26.6)	33.3 ± 27.1 (0.1–113)	35.6 ± 31.1 (3–113)
Mean estimated supplementary protein intake * (g/kg body weight per day)	0.23 ± 0.12 (0.08–0.37)	0.31 ± 0.35 (0–1.44)	0.36 ± 0.37 (0–1.32)
Users only: mean estimated supplementary protein intake * (g/kg body weight per day)	0.23 ± 0.12 (0.08–0.37)	0.42 ± 0.35 (0.0008–1.44)	0.42 ± 0.36 (0.04–1.32)
Mean estimated dietary protein intake in the general Danish male population (g/kg body weight per day)	1.36	1.27	1.21
Mean estimated protein intake from diet and supplements (g/kg body weight per day)	1.59 (1.44–1.74)	1.58 (1.27–2.71)	1.58 (1.21–2.54)
Users only: total mean estimated protein intake from diet and supplements (g/kg body weight per day)	1.59 (1.44–1.74)	1.69 (1.27–2.71)	1.63 (1.25–2.54)

* Data are presented as mean ± SD and (range).

**Table 4:** Reported adverse effects among fifteen participants able to recall the products most likely associated with the effects.

Age	Product Name	Reported Adverse Effect
18	PWO (Craze)	Insomnia
22	PWO (Super Pump)	Shaking, sweating, his eyes flickered a little
22	PWO (Animal)	Red skin, stinging
22	PWO (Jack 3D)	Shaking
29	PWO (Jack 3D)	Headache
19	PWO	Palpitations
26	PWO	Insomnia
23	PWO	Itching of the ears and back. Stinging for half an hour
22	Protein powder	Pimples on the back
19	Protein powder	Stomach rumble
34	* Glucose (High5)	Indigestion and too much sugar
24	Weight gainer	Chest pain
22	Beta-alanin	Tingling
35	Creatine	Headache
21 **	Protein shakes	Indigestion

Abbreviation: PWO: Pre-workout product. * Glucose: Product made of glucose. ** Diagnosed with lactose intolerance.

## Data Availability

Data from this paper can be made available upon request to the corresponding author.
